# Effect of the Mass Fraction of NiTi–TiB_2_ SHS-Particles on the Phase Composition, Structure, and Mechanical Properties of Inconel 625–NiTi–TiB_2_ Composites Produced by Direct Laser Deposition

**DOI:** 10.3390/ma15196861

**Published:** 2022-10-02

**Authors:** Alexey Matveev, Vladimir Promakhov, Nikita Schulz, Vladislav Bakhmat, Artem Babaev, Artem Semenov, Alexander Vorozhtsov

**Affiliations:** 1 Scientific and Educational Center “Additive Technologies”, National Research Tomsk State University, 634050 Tomsk, Russia; 2 “Nova-Health” Limited Liability Company, St. Visotsky, 28/7, 634040 Tomsk, Russia

**Keywords:** direct laser deposition, composite materials, ceramic particles, titanium diboride, phase composition, structure, hardness, mechanical strength

## Abstract

This paper studies the impact of the mass fraction of NiTi–TiB_2_ particles obtained by the method of self-propagating high-temperature synthesis (SHS) on the phase composition, structure, and mechanical properties of composites made by direct laser deposition from an Inconel 625–NiTiz–TiB_2_ powder mixture. Composites were obtained from a powder mixture with the mass fraction of particles at 5–10 wt%, and they consisted of an Inconel 625 metal matrix wherein ceramic inclusions of titanium diboride TiB_2_ were distributed. Increasing the mass fraction of SHS-produced NiTi particles from 30 to 95 wt% led to the emergence of a NiTi intermetallide phase in the matrix material as well as an increase in the average TiB_2_ particle size and formation of their agglomerates. In addition, an increase in the microhardness of the materials was observed. The graph of tensile strength of Inconel 625–NiTi–TiB_2_ samples has a parabolic shape with a maximum at 1000 MPa (when the mass fraction of SHS-produced NiTi–TiB_2_ particles is at 30 wt%). A further increase in the mass fraction of NiTi–TiB_2_ led to a decrease in the tensile strength down to 400 MPa. Here the deformation of samples decreases linearly as the ratio of composite particles in the initial mixture increases. From a comparative analysis of the results obtained, the optimal mass fraction of composite NiTi–TiB_2_ particles in the Inconel 625-NiTi–TiB_2_ powder mixture was found to be 5 wt%.

## 1. Introduction

In recent decades, additive manufacturing technologies have demonstrated explosive growth and have been globally appreciated both by industries and researchers [1]. Direct laser deposition is a specific case of additive manufacturing and allows for fabricating items with arbitrary geometry [2]. As compared to conventional sintering techniques (mold production, workpiece pressing, etc.), direct laser deposition reduces the involvement of human labor and logistics overhead to a minimum while shortening the preparation stages. [3]. This and other advantages shorten item fabrication time and reduce shop costs [4,5,6,7]. However, the development of modern industry requires not only the advancement of additive manufacturing technologies but also the enhancement of the quality of the powder raw stock used in item fabrication [8]. Most scientists and developers are focused on the creation of new powder materials for manufacturing items that would have advanced physicomechanical properties: strength, hardness, wear resistance, heat strength, and resistance to oxidation and aggressive media [9]. Such materials are in demand in different industries, including nuclear power, oil production, automotive, aerospace, and drive engineering [10]. As of today, the most popular powder materials used in the manufacturing of high-temperature parts by laser deposition are the Inconel family alloys [11]. However, despite the high physicomechanical properties of items fabricated by direct laser deposition, the improvement of their functionality and energy efficiency remains an open issue [12]. Composite materials with metal matrix and ceramic inclusions have advanced physicomechanical properties (high operating temperatures, wear resistance, mechanical strength, and hardness) as compared to conventional alloys, including those of the Inconel family [13,14,15,16]. The main approaches to obtaining composite powder materials (including those for use in AT installations) are methods for introducing ceramic particles into a metal melt. This approach has disadvantages associated with low wetting of ceramic particles by liquid metal. This leads to agglomeration and flotation of particles when introduced into the melt and, as a consequence, to the uneven distribution of particles inside the metal matrix [17,18]. In [19], a successful case of using an Inconel powder mixture with 625 + 5 wt% of NiTi–TiB_2_ for manufacturing materials by direct laser deposition was demonstrated. It should be noted that the NiTi–TiB_2_ powder was obtained by self-propagating high-temperature synthesis (SHS) based on exothermic reactions of the initial components of the NiB–Ti mixture [20]. In the course of SHS, an intermetallide NiTi matrix is obtained wherein TiB_2_ ceramic inclusions are uniformly distributed. Particles of the Inconel 625 + 5 wt% NiTi–TiB_2_ powder mixture were melted by the laser beam, and the ceramic inclusions of titanium diboride were wetted in the melt of the intermetallic NiTi matrix, which allowed them to be evenly distributed within liquid Inconel 625. The structure of the composites obtained by laser deposition inherited the structure of SHS particles and consisted of an Inconel 625 metal matrix wherein separate titanium diboride particles (TiB_2_) were uniformly distributed. The sizes of ceramic inclusions ranged between 0.05 and 1.2 µm, whereas their average size was 0.22 µm. The microhardness and tensile strength of the composites obtained were 402 HV_0.1_ and 920 MPa, respectively. These properties are clearly superior to those of the materials fabricated from pure Inconel (where microhardness and tensile strength were 273 HV_0.1_ and 850 MPa, respectively). The impact of the mass fraction of NiTi–TiB_2_ particles on the phase composition, structure, and mechanical properties of composites obtained by direct laser deposition from the Inconel 625–NiTi–TiB_2_ powder mixture remains an open issue. The purpose of this paper is to study the impact of the mass fraction of NiTi–TiB_2_ particles on the phase composition, structure, and mechanical properties of composites obtained by direct laser deposition from the Inconel 625–NiTi–TiB_2_ powder mixture.

## 2. Materials and Methods

Composite NiTi–TiB_2_ particles were obtained by self-propagating high-temperature synthesis from a NiB–Ti powder mixture. The synthesis methodology was provided in [20]. Powders of titanium (Ti) and nickel boron (NiB) were mixed in a stoichiometric ratio: 63.5 wt% NiB + 36.5 wt% Ti. Next, cylindrical samples were pressed from the resulting mixture. The synthesis process proceeded in a constant pressure reactor under an argon atmosphere. The reaction was initiated by local heating of the upper surface of the samples with a molybdenum coil. The resulting composites were ground to powder in a planetary mill. The particle size of NiTi–TiB_2_ SHS powder and Inconel powder varies from 50 to 150 µm and from 40 to 180 µm. The NiTi–TiB_2_ powder was mixed with a high-temperature Inconel 625 alloy powder. Six compositions with different mass fractions of NiTi-TiB_2_ particles were prepared: 5, 10, 30, 50, 70, and 90 wt%. The powder was mixed in a steel ball grinder for 20 min at a rotation frequency of 14 Hz. The Inconel 625–NiTi–TiB_2_ samples were obtained using an LS–3 fiber laser manufactured by IPG Photonics. The methodology and parameters of direct laser deposition are presented in Table 1 [19].

**Figure 1:**
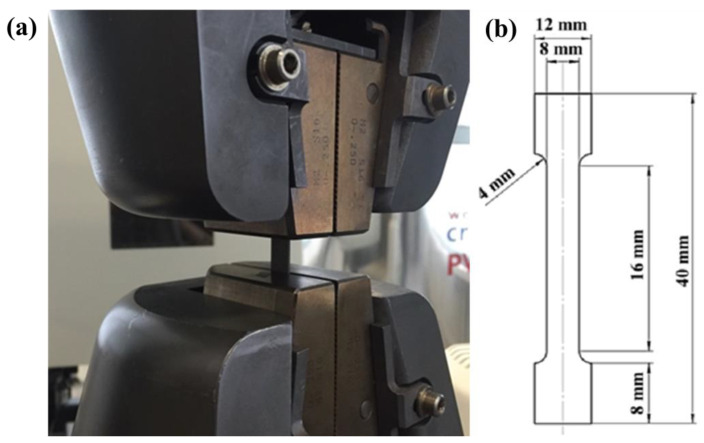
Instron universal static testing system (**a**), schematic diagram of the blade samples for tensile tests (**b**).

It should be noted that combined dual-side deposition was selected as the main strategy for fabricating composites from the Inconel 625–NiTi–TiB_2_ powder mixture. It is assumed that the selected strategy will allow for avoiding material shape distortion in the course of direct laser deposition. The phase composition of the materials obtained was investigated on a Shimadzu XRD-6000 diffractometer with Cu_ka_ radiation and a nickel-based filter (by Shimadzu Corporation, Tokyo, Japan). The phases were interpreted by comparing the peaks of the obtained diffraction patterns against the Powder Diffraction File 4 database of the International Center for Diffraction Data (ICDD). Calculations of the phase composition, lattice parameters, and coherent scattering region (CSR) sizes were carried out by refining the structure by the method of full profile analysis (the Rietveld method) [21,22]. The material structure was studied on a T-scan scanning electron microscope (SEM) with focused ion beams, and the microscope was fitted with an energy-dispersive X-ray spectrum (EDS) detector. The samples’ average microhardness was measured on a Buehler Wilson Micromet 6040 hardness meter (Buehler LLC, Lake Bluff, IL, USA) with Thixomet Pro image analysis software (Nikon Co., Tokyo, Japan). To measure the microhardness of composite materials obtained by direct laser growth by electroerosion processing, samples in the form of a disk 20 mm in diameter were cut from the central region of the composites. The measurements were carried out over the entire area of the samples (12 measurements for each area). Further, according to the results of measurements, the average value of the hardness of the composite material was determined. Tensile tests of the Inconel 625–NiTi–TiB_2_ materials were conducted on an Instron universal static testing system (by Illinois Tool Works Inc., Glenview, IL, USA), which is equipped with a video extensometer to accurately measure strain without the need for contact with the sample (Figure 1a). For the purpose of tensile testing, blade samples were fabricated from Inconel 625–NiTi–TiB_2_ composites by electroerosive processing. A schematic diagram of the blades is shown in Figure 1b. Tensile tests were carried out at room temperature. The strain rate of the samples was 2 mm per minute. For tensile tests, two samples were prepared for each composition.

## 3. Results and Discussion

### 3.1. The Impact of the Mass Fraction of NiTi–TiB_2_ Particles on the Phase Composition and Structure of Materials Obtained by Direct Laser Deposition from an Inconel 625–NiTi–TiB_2_ Powder Mixture

**Figure 2:**
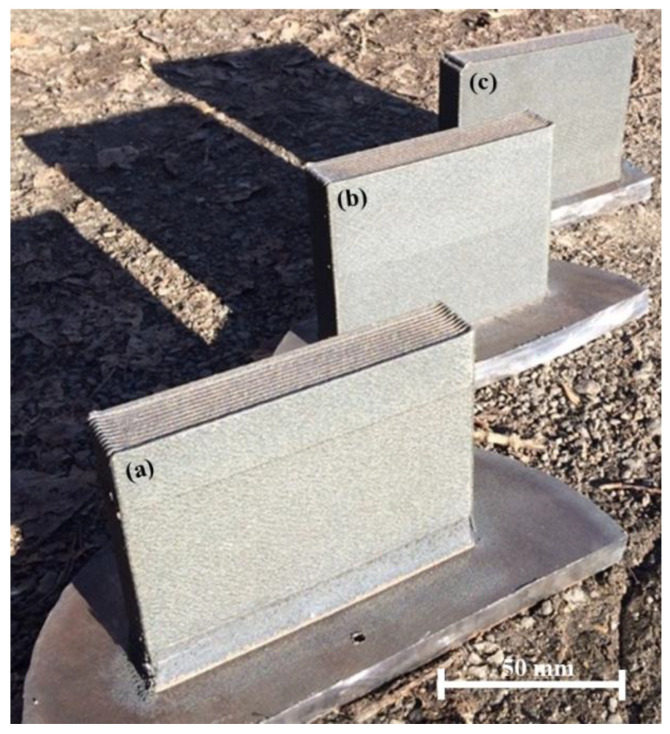
Appearance of materials obtained by direct laser deposition from a powder mixture of Inconel 625 + x wt% NiTi–TiB_2_: x = 5 (**a**), x = 50 (**b**), x = 90 (**c**).

Figure 2 shows the appearance of materials obtained by direct laser deposition from a Inconel 625–NiTi–TiB_2_ powder mixture. With the concurrent dual-side deposition strategy, it was possible to obtain rectangular materials that had a uniform structure of shell layers and did not have significant defects, regardless of the mass fraction of NiTi–TiB_2_ particles in the initial mixture.

**Figure 3:**
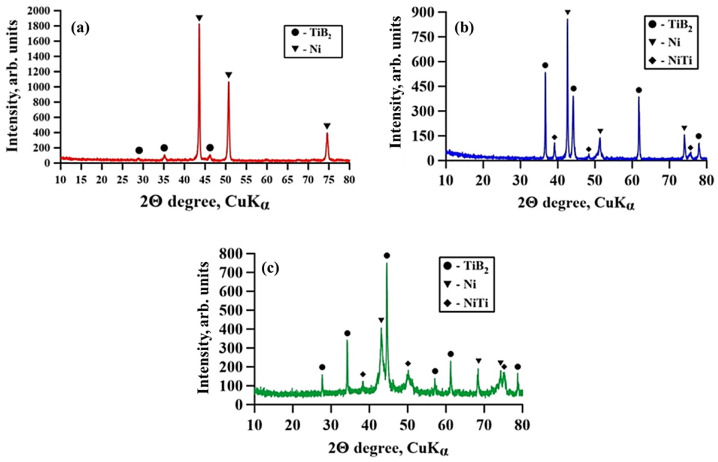
XRD images of composites obtained by direct laser deposition from a powder mixture of Inconel 625 + x wt% NiTi–TiB_2_: x = 5 (**a**), x = 50 (**b**), x = 90 (**c**).

Figure 3 shows XRD images of composites obtained by direct laser deposition from a Inconel 625–NiTi–TiB_2_ powder mixture. The results of the X-ray diffraction analysis of these materials are presented in Table 2.

**Figure 4:**
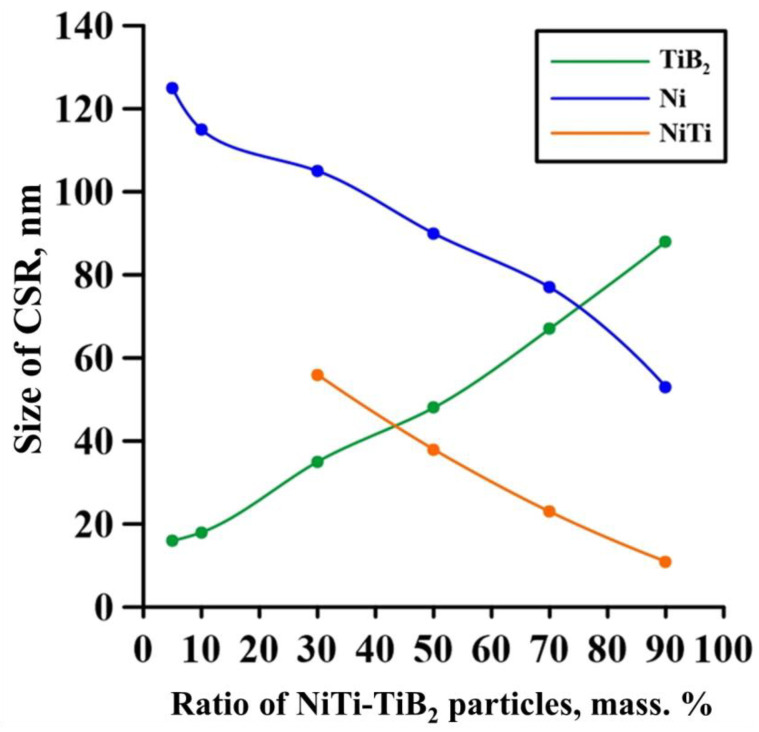
CSR size of the detected phases vs. the mass fraction of NiTi–TiB_2_ particles in the initial Inconel 625–NiTi–TiB_2_ mixture.

It was found that all composites contain a Ni phase that characterizes the Inconel 625 alloy as well as a TiB_2_ phase. As the mass fraction of NiTi–TiB_2_ particles in the initial mixture is increased from 5 to 90 wt%, an increase in the ratio of the titanium diboride phase from 4 to 49 wt% is observed, which is also accompanied by a decrease in the ratio of the nickel phase from 96 to 18 wt%. In the samples fabricated from powder mixtures with the mass fraction of NiTi–TiB_2_ at 30–90 wt%, a NiTi phase was found. As the mass fraction of composite NiTi–TiB_2_ particles was increasing, the mass fraction of the NiTi phase increased from 9 to 33 wt% as well. A comparison of the research findings with the XRD analysis of composite SHS-produced NiTi–TiB_2_ particles [20] has shown that in the materials obtained from the Inconel 625–NiTi–TiB_2_ powder mixture, the NiTi_2_ phase is not present, but it is found in SHS-produced NiTi-TiB_2_ particles. In the course of deposition, the NiTi_2_ phase fully dissolves under exposure to the laser beam, and the NiTi phase is partially dissolved in the Inconel 625 melt [23]. The lattice parameters of all the detected phases are close to the theoretical ones [24,25,26]. There is a slight shift of the peaks of these phases in the X-ray patterns. The shift of the peaks is explained by an increase in the content of the titanium diboride phase and a stronger effect of the laser on its particles, which can lead to a slight lattice distortion. Figure 4 shows a graph of the coherent scattering region (CSR) size of the detected phases vs. the mass fraction of NiTi–TiB_2_ particles in the initial Inconel 625–NiTi–TiB_2_ mixture.

It was found that as the mass fraction of NiTi–TiB_2_ particles in the initial Inconel 625–NiTi–TiB_2_ powder mixture is increased from 5 to 90 wt%, an increase in the CSR size of the TiB_2_ phase from 16 to 88 nm is observed and is accompanied by decreases in the CSR sizes of the Ni and NiTi phases from 125 to 53 and from 56 to 11 nm, respectively. The size of the coherent scattering region characterizes crystallite sizes in the materials [27]. An increase in the mass fraction of NiTi–TiB_2_ particles causes an increase in the content of titanium diboride particles and their specific surface in the initial mixture. Thanks to the high melting temperature, heat capacity, and heat conductivity of TiB_2_, its particles intensely absorb heat from the laser beam, which results in the growth of particle crystallites [28]. On the other hand, titanium diboride particles are efficient centers of crystallization for the melt of metals and intermetallides [29,30], which together with laser beam heat absorption leads to a considerable reduction in the growth of Ni and NiTi crystallites.

**Figure 5:**
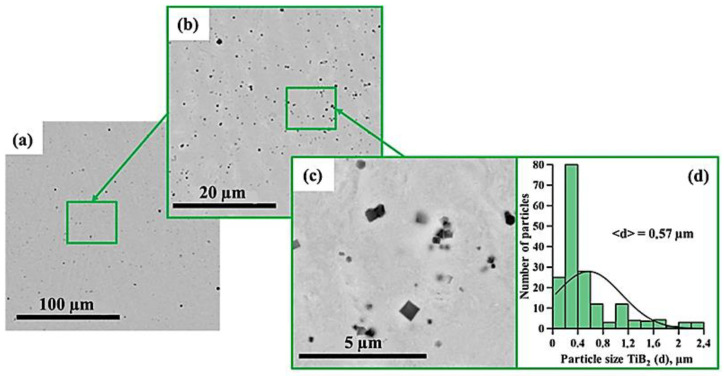
SEM image of the structure of a composite fabricated by direct laser deposition from the Inconel 625 + 5 wt% NiTi–TiB_2_ powder mixture (**a**–**c**), histogram of the distribution of ceramic inclusions by size (**d**).

**Figure 6:**
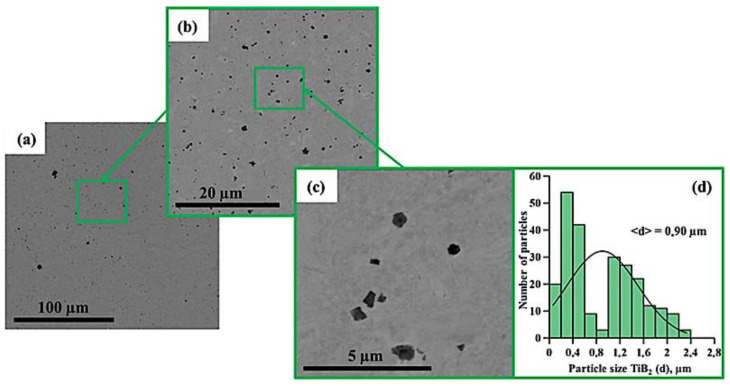
SEM image of the structure of a composite fabricated by direct laser deposition from the Inconel 625 + 10 wt% NiTi–TiB_2_ powder mixture (**a**–**c**), histogram of the distribution of ceramic inclusions by size (**d**).

**Figure 7:**
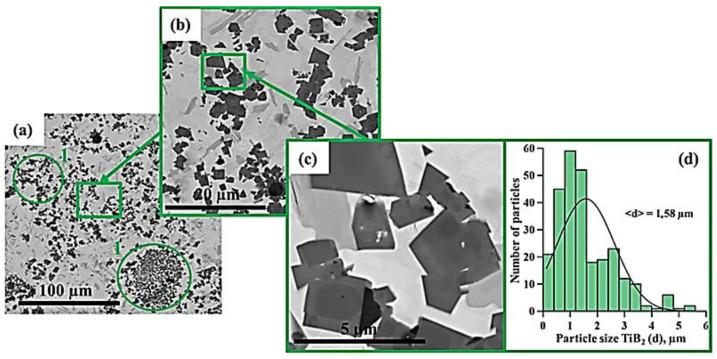
SEM image of the structure of a composite fabricated by direct laser deposition from the Inconel 625 + 30 wt% NiTi–TiB_2_ powder mixture (**a**–**c**), histogram of the distribution of ceramic inclusions by size (**d**).

**Figure 8:**
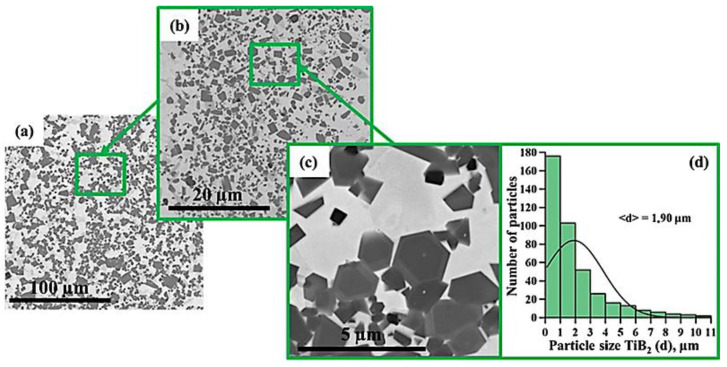
SEM image of the structure of a composite fabricated by direct laser deposition from the Inconel 625 + 50 wt% NiTi–TiB_2_ powder mixture (**a**–**c**), histogram of the distribution of ceramic inclusions by size (**d**).

**Figure 9:**
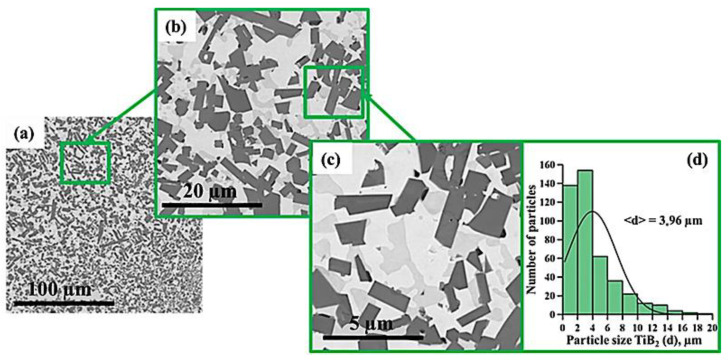
SEM image of the structure of a composite fabricated by direct laser deposition from the Inconel 625 + 70 wt% NiTi–TiB_2_ powder mixture (**a**–**c**), histogram of the distribution of ceramic inclusions by size (**d**).

**Figure 10:**
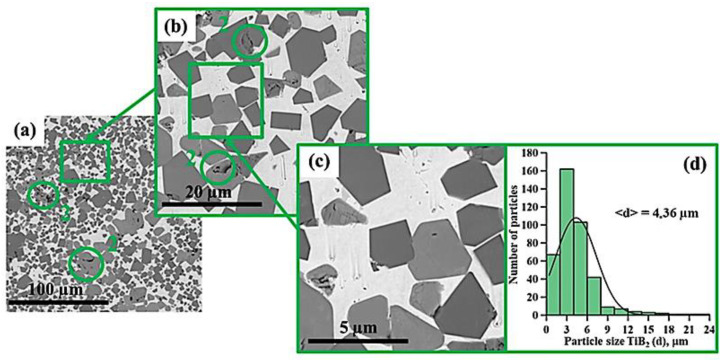
SEM image of the structure of a composite fabricated by direct laser deposition from the Inconel 625 + 90 wt% NiTi–TiB_2_ powder mixture (**a**–**c**), histogram of the distribution of ceramic inclusions by size (**d**).

**Figure 11:**
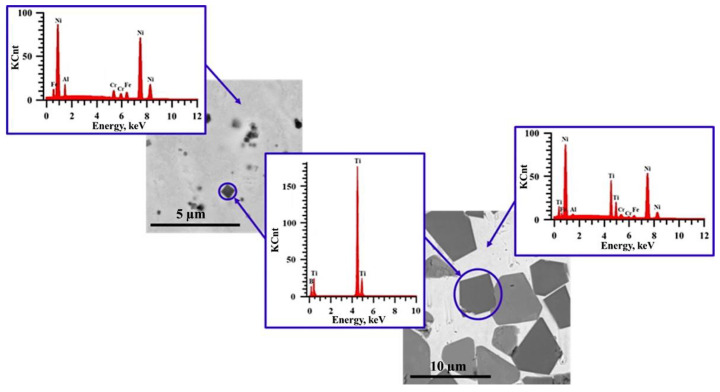
Qualitative elementary analysis of materials obtained by direct laser deposition from a 625–NiTi–TiB_2_ powder mixture.

Figure 5, Figure 6, Figure 7, Figure 8, Figure 9 and Figure 10 show SEM images of structures of composites fabricated by direct laser deposition from the Inconel 625–NiTi–TiB_2_ powder mixture. A qualitative elementary analysis of local regions in the structure of the materials is presented in Figure 11.

**Figure 12:**
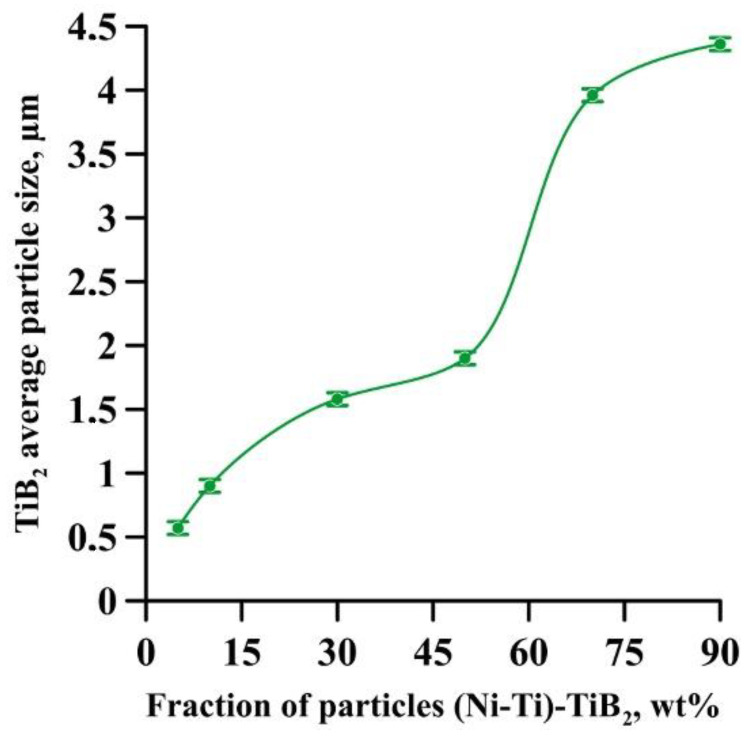
Graph of the average size of TiB_2_ particles in the Inconel 625–NiTi–TiB_2_ composite fabricated by direct laser deposition vs. the mass fraction of NiTi–TiB_2_ particles in the initial mixture.

It was found that the composites consist of irregularly shaped titanium diboride particles distributed within a nickel matrix. An elementary analysis of the matrix of the samples obtained from a mixture with the content of particles ranging from 5 to 10 wt% has shown the presence of nickel, chrome, iron, and aluminum, which is characteristic of Inconel 625 grade alloys. In the materials obtained from a mixture with the content of particles ranging from 30 to 90 wt%, the matrix contains titanium, which also suggests the presence of the NiTi phase in it. Materials obtained by direct laser deposition from the Inconel 625-–NiTi–TiB_2_ powder mixture inherit the structure of SHS-produced NiTi–TiB_2_ particles. However, the size of ceramic inclusions of titanium diboride and their distribution in the composite matrix are changed as the mass fraction of SHS particles in the initial mixture increases. Figure 12 shows a graph of the average size of TiB_2_ particles in the Inconel 625–NiTi–TiB_2_ composite vs. the mass fraction of NiTi–TiB_2_ particles in the initial mixture. The error value determination of the average particle size, taking into account the measurement method, was ±0.05 µm.

It was found that in composites obtained from the powder mixture with the mass fraction of 5 wt% NiTi–TiB_2_, titanium diboride particles are uniformly distributed within the metal matrix, and their average size is 0.57 µm. Increasing the mass fraction of SHS-produced NiTi–TiB_2_ particles from 2 to 10 wt% results in an increase in the average size of ceramic inclusions to 0.9 µm. Meanwhile, their uniform distribution is maintained. In the composites fabricated from a powder mixture with the mass fraction of NiTi–TiB_2_ at 30 wt%, the formation of titanium diboride particles agglomerates was detected, and their average size increased to 1.58 µm. In addition, in the structure of the composites, disruption in the uniform distribution of TiB_2_ particles and formation of local clusters of those particles (Figure 6, region 1) are observed. Clusters are formed because when the mass fraction of NiTi–TiB_2_ particles in the initial mixture is at 30 wt%, the proportion of ceramic inclusions of titanium diboride increases. This results in an increase in the absorbed heat from the matrix melt, a decrease in its temperature, and an increase in the solidification rate [31]. In this case, TiB_2_ particles do not have time to be uniformly distributed in the molten pool. An increase in the mass fraction of NiTi–TiB_2_ particles in the initial mixture from 30 to 90 wt% leads to intensive growth and fusion of ceramic inclusions, as well as the formation of large agglomerates. Here the average size of ceramic inclusions of TiB_2_ increases from 1.58 to 4.36 µm. The intensive growth of ceramic inclusions of titanium diboride is attributed to an increase in their content in the initial mixture. TiB_2_ particles intensely absorb heat from the laser beam and the matrix melt, and this induces the growth of TiB_2_ particle crystallites, which results in the growth of the particles [28,30]. In addition, an increase in the mass fraction of titanium diboride particles and a decrease in the concentration of the Inconel 625 powder lead to closer interactions of ceramic inclusions with each other. Exposure to laser beam radiation triggers processes of recrystallization of ceramic particles, and this leads to the formation of large agglomerates [32]. In the sample fabricated from a powder mixture with the mass fraction of NiTi–TiB_2_ particles at 50–90 wt%, no local clusters of ceramic inclusions were observed, and this is also attributed to a high mass fraction of NiTi–TiB_2_ particles in the initial mixture. Here the layer of Inconel 625 melt is thinner as compared to materials obtained from a powder mixture with the SHS particle mass fraction at 30 wt%. A thinner layer of the melt allows TiB_2_ particles to be distributed in its molten pool before solidification processes have taken place while also forming a more uniform matrix structure. It should be noted that a change in the concentration of the composite SHS powder NiTi–TiB_2_ in the initial mixture of Inconel 625 + 5 wt% NiTi–TiB_2_ leads to a change in the size distribution of titanium diboride ceramic inclusions (Figure 4, Figure 5, Figure 6, Figure 7, Figure 8 and Figure 9d). The size of ceramic inclusions in materials obtained by the AT method from a mixture of Inconel 625 + 5 wt% NiTi–TiB_2_ is comparable to the size of titanium diboride particles in the NiTi–TiB_2_ SHS composite powder. With an increase in the proportion of composite SHS powder NiTi–TiB_2_, small ceramic inclusions are recrystallized under the action of a laser beam, which leads to the formation of larger inclusions. So, for example, with an increase in the concentration of NiTi–TiB_2_ composite particles up to 50 wt%, small inclusions of titanium diboride undergo recrystallization under the influence of a laser beam and form larger inclusions, the size of which is close to 1 μm. The formation of these particles leads to an increase in their concentration in the material and, consequently, an increase in their contribution to the overall size distribution. A similar effect is also observed with an increase in the proportion of NiTi–TiB_2_ to 70 and 90 wt%. Small inclusions of titanium diboride interact with each other more tightly and undergo recrystallization under the influence of the laser beam, which leads to the formation of larger particles up to 4 μm in size. Increasing the concentration of ceramic inclusions of this size also increases their contribution to the overall size distribution. It should be noted that in samples with 5–50 wt%, there were no defects and pores at the boundaries between the ceramic particles and the matrix, which indicates good wettability of titanium diboride particles by the matrix melt during material deposition. Most defects and pores were observed in the agglomerates of materials obtained with a mass fraction of 90 wt% (Figure 9, region 2). The presented results demonstrate the high efficiency of using NiTi–TiB_2_ composite SHS powders as an additive to the main powder raw material for obtaining materials by laser growth. The obtained data can be compared with the work [33] where the authors obtained Al–SiC composite materials by sintering a powder mixture of Al-10 vol.% SiC (8 and 44 µm). The sintered composites consisted of silicon carbide particles that were dispersed in an aluminum matrix. An analysis of the structure of the materials showed that there are pronounced defects and pores at the interface between the matrix and particles. The paper reports that the use of silicon carbide particles with a size of 8 μm leads to agglomeration and their uneven distribution during sintering. Kim S. et al. [33] solved this problem by coating silicon carbide particles with a layer of copper. The coating was carried out using the method of inductively coupled plasma (ICP). The authors found that the coating of ceramic particles with copper made it possible to increase their wettability. This led to a decrease in pores and defects in the structure of composite materials obtained by sintering. Thus, Kim S. et al. [33] demonstrated the effectiveness of the use of composite metal matrix particles in the production of bulk materials. The application of the SHS method makes it possible to obtain metal matrix particles without the use of external energy sources, but due to exothermic reactions of the initial components of the mixture: NiB and Ti [20]. During the reaction, particles of titanium diboride are formed, which are surrounded by a Ni–Ti intermetallic melt. The formation of a matrix melt prevents the processes of agglomeration and recrystallization, which makes it possible to obtain isolated particles with an average size of 0.57 μm.

### 3.2. The Impact of the Mass Fraction of Niti-Tib_2_ Particles on the Mechanical Properties of Materials Obtained by Direct Laser Deposition from an Inconel 625–NiTi–TiB_2_ Powder Mixture

**Figure 13:**
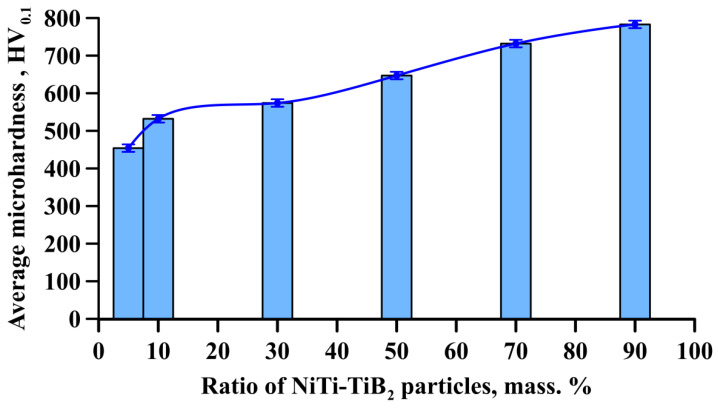
Average microhardness of samples obtained by direct laser deposition from an Inconel 625–NiTi–TiB_2_ powder mixture vs. the mass fraction of NiTi–TiB_2_ particles.

Figure 13 shows a graph of the average microhardness of samples obtained by direct laser deposition from an Inconel 625–NiTi–TiB_2_ powder mixture vs. the mass fraction of composite NiTi–TiB_2_ particles. It was found that as the mass fraction of NiTi–TiB_2_ in the initial mixture is increased from 5 to 90 wt%, an increase in the average microhardness of the grown samples from 460 to 790 HV_0.1_ is observed. The increase in the average microhardness of the samples is attributed to the high hardness of titanium diboride (25–35 GPa) [34]. An increase in the mass fraction of NiTi–TiB_2_ particles leads to an increase in the mass and volume fractions of ceramic inclusions, which integrally leads to an increase in the hardness of Inconel 625–NiTi–TiB_2_ composites [35].

**Figure 14:**
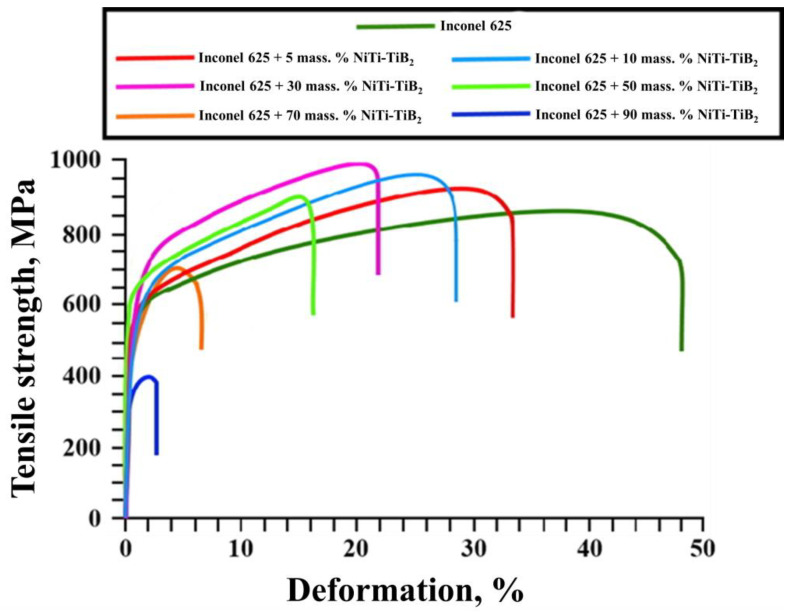
Stress–strain diagrams obtained during tensile tests of Inconel 625–NiTi–TiB_2_ samples with the mass fraction of NiTi–TiB_2_ particles at 5–90 wt%.

**Figure 15:**
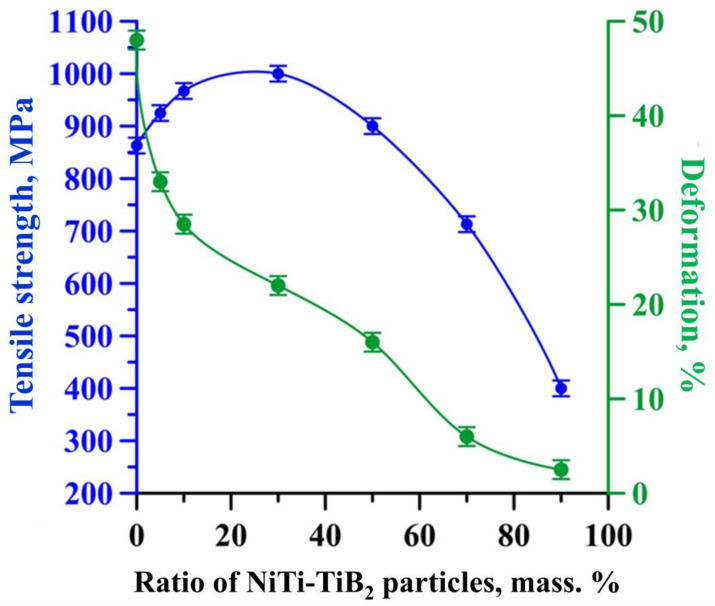
Dependency of the tensile strength and tensile deformation of samples obtained by direct laser deposition from an Inconel 625–NiTi–TiB_2_ powder mixture vs. the mass fraction of NiTi–TiB_2_ particles.

Figure 14 shows typical stress–strain diagrams obtained during tensile tests of Inconel 625–NiTi–TiB_2_ samples with the mass fraction of NiTi–TiB_2_ particles at 5–95 wt%. Figure 15 shows a graph of the dependency of the tensile strength and tensile deformation on the mass fraction of NiTi–TiB_2_ particles.

The graph showing the dependency of the tensile strength of Inconel 625–NiTi–TiB_2_ samples during tensile tests vs. the mass fraction of NiTi–TiB_2_ particles in the initial mixture is parabolic. An increase in the mass fraction of NiTi–TiB_2_ particles in the initial mixture from 0 to 30 wt% results in an increase in the tensile strength from 860 to 1000 MPa. However, further increases in the mass fraction of particles from 30 to 90 wt% result in a decrease in the tensile strength down to 400 MPa. Here the deformation of samples decreases linearly from 48 to 2.5% as the mass fraction of composite NiTi–TiB_2_ particles in the initial mixture is increased from 0 to 90 wt%. An increase in the tensile strength of composite materials obtained from a powder mixture with the mass fraction of NiTi–TiB_2_ particles at 5–30 wt% is attributed to the mechanism of heterophase reinforcement of the metal matrix with ceramic inclusions [19]. In addition, in the course of laser deposition, titanium diboride particles act as centers of crystallization of matrix material grains, which leads to a decrease in their average size and therefore an increase in the samples’ strength [36,37,38]. However, as the mass fraction of SHS-produced NiTi-TiB_2_ particles in the structure of the composites is increased from 30 to 90 wt%, thermal effects start to cause internal stresses at the particle–matrix interfaces [39]. These stresses promote the formation of cracks and defects, which leads to a decrease in the composite strength. In addition, pores are observed in the structure of materials in the region of recrystallization of titanium diboride particles. This leads to a breach of this region’s integrity and a decrease in the sample strength [40]. From a comparative analysis of the research findings, an optimal mass fraction of composite NiTi–TiB_2_ particles in the Inconel 625–NiTi–TiB_2_ powder mixture was determined. It was found that for the particle mass fraction of 5–10 wt%, the structure of the materials obtained by selective laser deposition has a uniform distribution of individual particles of titanium diboride within the matrix material while particle agglomerates are not formed. No defects and pores were found in the structure of the materials, which indicates good wetting of the particles by the matrix melt. It was shown that materials obtained from the mixture of Inconel 625 and 5–10 wt% NiTi–TiB_2_ have an optimal combination of hardness and strength while maintaining a rather high plasticity. Table 3 shows a comparison of the mechanical properties of materials Inconel 625 + 5 wt% NiTi–TiB_2_ and Inconel 625 +10 wt% NiTi–TiB_2_ with data from [41].

A comparison of the results showed that the hardness of materials obtained from powder mixtures of Inconel 625 with 5 and 10 wt% NiTi–TiB_2_ is higher than that of materials obtained from pure Inconel 625 [41] by 40 and 48%, respectively. In addition, the addition of SHS composite particles leads to an increase in the ultimate strength by 20 and 25%, respectively. On the other hand, there is a decrease in deformation by 16 and 21%, respectively. Thus, the analysis carried out indicates a high efficiency of using the Inconel 625 + 5–10 wt% NiTi–TiB_2_ powder mixtures for the production of composite materials by direct laser growth.

## 4. Conclusions

The studies performed for the purpose of this research have shown that an increase in the mass fraction of SHS-produced NiTi–TiB_2_ particles in the Inconel 625-NiTi-TiB_2_ powder mixture leads to a change in the phase composition, structure, and mechanical properties of the materials obtained by direct laser deposition. From a comparative analysis of the research findings, the optimal mass fraction of composite NiTi–TiB_2_ particles in the Inconel 625–NiTi–TiB_2_ powder mixture was determined to be 5–10 wt%. The results obtained demonstrate the prospect of using composite materials obtained by the method of self-propagating high-temperature synthesis as additives to powder materials used in direct laser growth installations. The use of composite metal matrix powders for the production of products (e.g., turbine blades of a gas turbine engine) by laser growth makes it possible to increase their mechanical properties (compared to traditional alloys) while maintaining or reducing weight.

## Figures and Tables

**Table 1:** Direct laser growth parameters [17].

Beam Diameter in the Treatment Area, mm	Power, W	Side Bead Deposition Rate, mm/s	Intermediate Bead Deposition Rate, mm/s	Powder Consumption, g/min	X Offset, mm	Z Offset, mm
1.5	500	10	15	5.1	0.7	0.2

**Table 2:** X-ray diffraction analysis results for composites obtained by direct laser deposition from a Inconel 625–NiTi–TiB_2_ powder mixture.

Sample of Inconel 625 + x wt% NiTi–TiB_2_	Detected Phases	Phase Content, Mass %	Lattice Parameters, Ǻ	CSR Size, nm
x = 5	Ni	96	a = 3.5241	125
	TiB_2_	<4	a = 2.9468	16
			c = 3.1359	
x = 10	Ni	94	a = 3.5239	115
	TiB_2_	6	a = 2.9765	18
			c = 3.1989	
x = 30	Ni	75	a = 3.5241	105
	TiB_2_	16	a = 3.0234	35
			c = 3.2202	
	NiTi	9	a = 2.9990	56
x = 50	Ni	56	a = 3.5236	90
	TiB_2_	27	a = 3.0233	48
			c = 3.2205	
	NiTi	17	a = 3.0111	38
x = 70	Ni	37	a = 3.5239	77
	TiB_2_	38	a = 3.0238	67
			c = 3.2208	
	NiTi	25	a = 3.0113	23
x = 90	Ni	18	a = 3.5231	53
	TiB_2_	49	a = 3.0236	88
			c = 3.2205	
	NiTi	33	a = 3.0118	11

**Table 3:** Mechanical properties of materials Inconel 625 + 5 wt% NiTi-TiB2, Inconel 625 + 10 wt% NiTi-TiB_2_, and Inconel 625 [41].

Properties	Inconel 625 + 5 wt% NiTi–TiB_2_	Inconel 625 + 10 wt% NiTi–TiB_2_	Inconel 625 [41]
Microhardness	460 HV	532 HV	275 HV
Tensile strength	930 MPa	970 MPa	721 MPa
Deformation	33%	28%	49%

## Data Availability

Not applicable.
